# In Vitro Model of Vascularized Bone: Synergizing Vascular Development and Osteogenesis

**DOI:** 10.1371/journal.pone.0028352

**Published:** 2011-12-02

**Authors:** Cristina Correia, Warren L. Grayson, Miri Park, Daphne Hutton, Bin Zhou, X. Edward Guo, Laura Niklason, Rui A. Sousa, Rui L. Reis, Gordana Vunjak-Novakovic

**Affiliations:** 1 Department of Biomedical Engineering, Columbia University, New York, New York, United States of America; 2 Department of Biomedical Engineering, Johns Hopkins University, Baltimore, Maryland, United States of America; 3 Department of Biomedical Engineering, Yale University, New Haven, Connecticut, United States of America; 4 3B's Research Group – Biomaterials, Biodegradables and Biomimetics, University of Minho, Guimarães, Portugal; Universidade do Porto, Portugal

## Abstract

Tissue engineering provides unique opportunities for regenerating diseased or damaged tissues using cells obtained from tissue biopsies. Tissue engineered grafts can also be used as high fidelity models to probe cellular and molecular interactions underlying developmental processes. In this study, we co-cultured human umbilical vein endothelial cells (HUVECs) and human mesenchymal stem cells (MSCs) under various environmental conditions to elicit synergistic interactions leading to the colocalized development of capillary-like and bone-like tissues. Cells were encapsulated at the 1∶1 ratio in fibrin gel to screen compositions of endothelial growth medium (EGM) and osteogenic medium (OM). It was determined that, to form both tissues, co-cultures should first be supplied with EGM followed by a 1∶1 cocktail of the two media types containing bone morphogenetic protein-2. Subsequent studies of HUVECs and MSCs cultured in decellularized, trabecular bone scaffolds for 6 weeks assessed the effects on tissue construct of both temporal variations in growth-factor availability and addition of fresh cells. The resulting grafts were implanted subcutaneously into nude mice to determine the phenotype stability and functionality of engineered vessels. Two important findings resulted from these studies: (*i*) vascular development needs to be induced prior to osteogenesis, and (*ii*) the addition of additional hMSCs at the osteogenic induction stage improves both tissue outcomes, as shown by increased bone volume fraction, osteoid deposition, close proximity of bone proteins to vascular networks, and anastomosis of vascular networks with the host vasculature. Interestingly, these observations compare well with what has been described for native development. We propose that our cultivation system can mimic various aspects of endothelial cell – osteogenic precursor interactions *in vivo*, and could find utility as a model for studies of heterotypic cellular interactions that couple blood vessel formation with osteogenesis.

## Introduction

In native bone, synergistic interactions between osteoblasts/osteogenic precursors and endothelial cells enable coordinated development of vasculature and mineralized tissue. In the process of intramembranous ossification during craniofacial bone growth, this cell coupling results in close spatial relationships between the two tissues in newly forming bone, with the vascular network serving as a ‘template’ for bone mineral deposition [1]. A synergy between the two cell populations has also been observed during endochondral ossification. A murine model was used to demonstrate that when the HIF-1α protein was constitutively activated in osteoblasts by conditional deletion of the *Vhl* gene, the result was higher vascularity in long bones with complementary increases in bone volumes [2]. In a recent study, it was shown that osteoblast precursors occupy pericytic locations as they invade the cartilage template along with blood vessels to form new trabecular bone during ossification of long bones [3]. Still, many of the mechanisms guiding interactions between endothelial cells and osteogenic precursors remain largely unknown due to the complexity of the *in vivo* environment.

The need to vascularize tissue engineered bone grafts, during culture and following implantation, has led to studies between endothelial cells and osteoblasts/osteo-progenitors [4], [5], [6], [7]. Interestingly, despite the preponderance of evidence linking vascular development and osteogenesis *in vivo*, it has been decidedly difficult to simultaneously form capillary-like networks and mineralized deposits *in vitro*, within a single culture environment. Whereas beneficial effects of osteogenic precursors on vascular network formation have been demonstrated [7], [8], adequate formation of bone-like tissue within the same constructs has not been achieved. The effect of endothelial population on osteogenesis remains conflicting: some studies have indicated a positive effect of endothelial cells on mineral deposition [6], [9], [10], [11], [12], while other studies have described molecular pathways via which endothelial cells inhibited osteogenesis [13], [14].

Recently, one group circumvented the difficulty of inducing adequate vascular development and osteogenic differentiation by seeding mesenchymal stem cells (MSCs) into polymer scaffolds and inducing osteogenesis prior to seeding MSCs and human umbilical vein endothelial cells (HUVECs) into a gel substrate [15]. When these constructs were implanted subcutaneously into the dorsum of nude mice, developed vascular networks anastomosed to the host vasculature, while after eight weeks of *in vivo* cultivation evidence of mineral deposition was revealed. Several other groups have shown that implanting biomaterial constructs with a mixture of mesenchymal and vascular or hematopoietic progenitor cells enabled the development of vascularized tissues *in vivo* [12], [16], [17], [18]. However, realistic *in vitro* cultivation models are required to elucidate the mechanistic interactions of both cell populations during the formation of vascularized bone.

**Figure 1:**
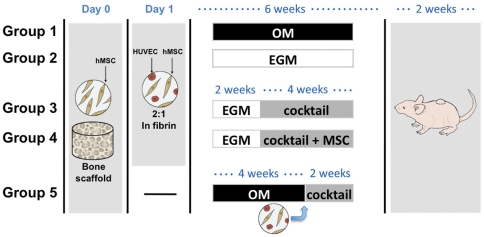
Schematic of experimental approaches. Groups 1 & 2 are ‘controls’ where constructs were provided osteogenic supplements (**OM**) or endothelial factors (**EGM**) for 6 weeks. In Groups 3 & 4, vascular differentiation was induced for 2 weeks before adding osteogenic factors in a cocktail medium (EGM+OM at 1∶1 ratio). No additional cells were added at this point in Group 3 (**EGM|cocktail**), while osteo-induced MSCs were seeded into the pore spaces in Group 4 (**EGM|cocktail+MSCs**). These were compared with cultures in Group 5 where only MSCs were added initially and cultured in OM for 4 weeks. A co-culture of HUVECs and MSCs were then added and constructs cultured in cocktail medium (**OM|cocktail**) for remaining 2 weeks. Constructs of all groups were implaneted sub-cutaneously in nude mice for additional 2 weeks.

In this study, we hypothesize that the sequential application of growth factors, to firstly induce the formation of stable vasculature and subsequently initiate osteogenic differentiation, could provide a biologically-inspired *in vitro* model of bone vascularization. HUVECs and human MSCs were cultured in decellularized trabecular bone constructs using fibrin as a cell carrier to provide an environment conducive to the formation of capillary-like networks. Coordinated development of the two tissue compartments was evaluated over a two-stage procedure (6 weeks *in vitro* culture followed by a 2 week sub-cutaneous implantation), to establish an alternative model for engineering bone-like constructs containing vascular networks (**Fig. 1**).

## Materials and Methods

### Materials

Fetal bovine serum (FBS), Dulbecco's Modified Eagle Medium (DMEM), Penicillin–Streptomycin (Pen–Strep), HEPES and trypsin/EDTA were obtained from Invitrogen (Carlsbad, CA). Ascorbic acid-2-phosphate, dexamethasone, sodium-β-glycerophosphate, Triton X-100, fibrinogen and thrombin were obtained from Sigma-Aldrich (St. Louis, MO). Basic fibroblast growth factor (bFGF) and bone morphogenetic protein 2 (**BMP2**) were obtained from Peprotech (Rocky Hill, NJ). Endothelial Growth Medium-2 (**EGM**) was obtained from Lonza (Walkersville, MD). Phosphate buffered saline (PBS) and proteinase K were obtained from Fisher Scientific (Pittsburgh, PA). All other substances were of analytical or pharmaceutical grade and obtained from Sigma-Aldrich.

### Human Mesenchymal Stem Cells (MSCs)

Bone marrow-derived human mesenchymal stem cells (MSCs) were isolated from the mononuclear fraction of a bone marrow aspirate obtained from a commercial source (Cambrex, CA) based on their attachment to tissue culture plastics [19], expanded in high glucose DMEM supplemented with 10% FBS, 1% Pen–Strep and 1 ng/mL basic fibroblast growth factor (bFGF) to the 3^rd^ passage. These cells have been characterized for their ability to differentiate into osteogenic, chondrogenic, and adipogenic phenotypes *in vitro* and for their ability to give rise to new bone formation *in vitro* and *in vivo* [19], [20], [21]. Three independent series of experiments were performed, each with triplicates of samples for each experimental group, data point and analytical method.

### Human umbilical vein endothelial cells (HUVECs)

Fresh umbilical veins were obtained from the neonatal unit at Columbia University following an approved IRB protocol (IRBAAAC4839). To isolate HUVECs, the vein was flushed with HEPES buffer to remove residual blood and treated with trypsin for 15 minutes to remove the endothelial cells. Cells were flushed from the vein with HEPES and collected in centrifuge tubes. The suspension was centrifuged at 300 g for 5 minutes and the supernatant was discarded. Cells were resuspended in EGM and cultured in tissue culture flasks. Non-adherent cells were washed out after 1 day. When the HUVECs grew to confluence, they were trypsinized, counted and cryopreserved.

### Medium screening studies

In this study, HUVECs were expanded in EGM to the 4^th^ passage. HUVECs used in preliminary screening studies were suspended in serum-free medium at 1×10^6^ cells/mL and then incubated with 10 ml/mL of Vybrant DiI (Molecular Probes) for 30 minutes at 37°C. The cells were washed of excess dye three times prior to mixing with hMSCs in a 1∶1 ratio, and encapsulation in fibrin hydrogel in the wells of a 96-well plate and cultured for 4 weeks *in vitro*. A 1∶1 ratio was chosen in pilot studies (data not shown) as it provided robust and stable vascular networks while enabling bone formation. The hydrogels were divided into five groups depending on the various growth medium provided the constructs. These included osteogenic medium (OM) containing low glucose DMEM, 10% FBS, 1% Pen–Strep supplemented with osteogenic factors: 100 nM dexamethasone, 10 mM sodium-β-glycerophosphate, and 50 µg/mL ascorbic acid-2-phosphate. The other groups were provided EGM or a cocktail of OM and EGM in a 1∶1 ratio. The temporal sequence was investigated by providing the cells EGM only for 2 weeks followed by cocktail for the subsequent two weeks or cocktail medium supplemented with 10 ng/ml of BMP-2. The development of vascular structures was imaged with a Zeiss 510 confocal microscope using an image stack 200 µm thick with 10 µm spacing between slices.

### Decellularized bone scaffolds

Bone plugs (4 mm diameter×2 mm high) were prepared as in our previous studies [22]. Briefly, trabecular bone was cored from the subchondral region of carpometacarpal joints of 2-week to 4-month old bovine calves, washed with high velocity stream of water to remove the marrow from the pore spaces, followed by sequential washes in PBS, hypotonic buffer, detergent and enzymatic solution to remove any remaining cellular material. At the end of the process, decellularized bone plugs were rinsed repeatedly in PBS, freeze-dried and cut into scaffolds for cell cultivation. The dry weight and exact length of each plug was measured and used to calculate the scaffold density and porosity. Scaffolds were sterilized in 70% ethanol for one day and incubated in culture medium overnight prior to cell seeding.

### Cell encapsulation and seeding

To induce bone formation, 20 µl suspension of MSCs at a density of 10×10^6^ cells/mL was seeded into blot-dried scaffolds, divided into five groups, and incubated overnight to allow cell attachment as shown in **Fig. 1**. At day 1, the solutions of fibrinogen (5 mg/mL) and thrombin (10 Units/mL) were prepared. In Groups 1–4, HUVECs and MSCs, intended to form stable vasculature network, were encapsulated in fibrin at a 2∶1 ratio and a density of 30×10^6^ cells/mL. Thrombin was added to crosslink the gel, giving a final fibrin concentration of 4 mg/mL. Before crosslinking occurred, 20 µl of cell/gel suspension was pipetted into blot-dried scaffolds to allow uniform cell seeding throughout the scaffolds. Before the gels became fully cross-linked, they were aspirated under a light vacuum so that the fibrin gel coated the walls of the scaffolds, but did not fill the pore spaces. In Group 5, this same step was performed on MSC-seeded scaffolds after 4 weeks of osteogenic culture.

### In vitro culture

Five experimental groups were established to study vascularized bone constructs, including three main groups and two controls (**Fig. 1**). **Group 1** (control for osteogenic conditions) utilized osteogenic medium (**OM**) throughout the culture, containing low glucose DMEM, 10% FBS, 1% Pen–Strep supplemented with osteogenic factors: 100 nM dexamethasone, 10 mM sodium-β-glycerophosphate, and 50 µg/mL ascorbic acid-2-phosphate, supplemented with BMP-2 (at 10 ng/mL) for 6 weeks. **Group 2** (control for vasculogenic conditions) utilized endothelial growth medium (**EGM**) throughout the culture. **Group 3** utilized EGM for 2 weeks and then a cocktail medium composed by EGM and OM at 1∶1 ratio (**EGM|cocktail**) for 4 weeks. **Group 4** was designed exactly as Group 3 except that osteo-induced MSCs were added into scaffold pore spaces at the 2-week time point (**EGM|cocktail+MSC**). In **Group 5**, MSCs only were seeded into the scaffolds and cultured in osteogenic medium for 4 weeks, at which point HUVECs and MSCs in fibrin were added to the constructs and cultured in cocktail medium for an additional 2 weeks (**OM|cocktail**).

### In vivo implantation

After 6 weeks of *in vitro* culture, constructs of all groups were evaluated for 2 weeks in a subcutaneous implantation nude mouse model *in vivo*. NOD SCID mice (NOD.CB17-*Prkdc^scid^*/NCrHsd, Harlan) were anaesthetized by a subcutaneous injection of ketamine (80–100 mg/kg) and xylazine (5–10 mg/kg). Analgesia was provided with buprenorphine (0.05–0.1 mg/kg). Constructs were implanted into separate subcutaneous dorsal pockets (one construct per pocket, two pockets per animal) according to an approved Columbia University Institutional Animal Care and Use Committee (IACUC) protocol. Implants were retrieved after 2 weeks for analysis of blood vessel anastomosis and bone growth.

### DNA assay

Constructs were washed in PBS, cut in half, placed into micro-centrifuge tubes and incubated overnight at 56°C in 1 mL of digestion buffer (10 mM Tris, 1 mM EDTA and 0.1% Triton X-100) containing 0.1 mg/mL proteinase K. The supernatants were collected and diluted 10 times to bring into the linear range of the Picogreen assay. A standard curve was prepared from a solution of bacteriophage λ DNA obtained from Molecular Probes. Picogreen dye (Molecular Probes, OR) was added to the samples in 96-well plates (100 µL dye into each 100 µL sample) and read in a fluorescent plate reader (excitation 485 nm, emission 528 nm).

### Live-Dead assay

Constructs were cut in half, washed in PBS, incubated with calcein AM (indicating live cells) and ethidium homodimer-1 (indicating dead cells) according to manufacturer's protocol (LIVE/DEAD (R) Viability/Cytotoxicity Kit, Molecular Probes) and imaged on a confocal microscope. Optical slices were taken from the surface at 10 µm intervals, up to the depth of 160 µm and presented as a vertical projection.

### Immunohistochemistry

Constructs were washed in PBS and fixed in 10% formalin for 1 day. Samples were then decalcified for 2 days with Immunocal solution, dehydrated in graded ethanol washes, embedded in paraffin, sectioned to 5 µm and mounted on glass slides. Sections were deparaffinized with Citrisolv, rehydrated with a graded series of ethanol washes, blocked with normal serum, and stained with primary antibodies followed by the secondary antibody and development with a biotin/avidin system. Immunohistochemistry was performed for anti-human collagen I (Abcam ab6308), bone sialoprotein II (BSP) (Chemicon AB1854), CD31 (Millipore 04-1074) and von Willebrand factor (vWF) (Sigma-Aldrich F3520). Counterstaining was performed with hematoxylin. The serum, secondary antibody and developing reagents were obtained from Vector Laboratories and included in the Vector Elite ABC kit (universal) (PK6200) and DAB/Ni Substrate kit (SK-4100). Negative controls were performed by omitting the primary antibody incubation step.

### Micro Computerized Tomography (μCT)

μCT was performed on all five cell-seeded groups after 6 weeks of *in vitro* culture. As a control, unseeded scaffolds were remained in OM throughout culturing period and analyzed at this time point. A modification of a previously developed protocol was used [23]. The samples were aligned along their axial direction and stabilized in a 2 mL centrifuge tube that was clamped within the specimen holder of a vivaCT 40 system (SCANCO Medical AG, Basserdorf, Switzerland). The 2 mm length of the scaffold was scanned at a 21 µm isotropic voxel size. The total bone volume (BV), accounting for the sum of the bone matrix in the scaffold and the new mineralized bone, was obtained from the application of a global thresholding technique so that only the mineralized tissue is detected. The bone volume fraction (BV/TV) was calculated as the ratio of the BV and the total volume (TV) of the sample. Spatial resolution of this full voxel model was considered sufficient for evaluating the micro-architecture of the samples.

### Evaluation of in vivo anastomosis and new bone formation

After 2 weeks of subcutaneous implantation in NOD SCID mice, samples were harvested, washed in PBS and macroscopic photographs were taken with the stereomicroscope. Samples were cut in half, and used for soft and hard tissue histology. Anastomosis of *in vitro* developed capillary networks with host vasculature was evaluated by immunolocalization of human CD31 (Millipore 04-1074) as described above. Counterstaining with hematoxylin was performed to detect mouse red blood cells within human capillary lumen. New bone formation was evaluated on undecalcified sections processed according to hard tissue histology methods: constructs were fixed in 10% formalin for 1 day and dehydrated with sequential washes in 70% ethanol (2 days), 100% ethanol (2 days with twice daily solution changes), and toluene (2 days with once daily solution change). Constructs were then washed in activated methyl methacrylate (MMA) with daily changes of MMA solution for four days at 4°C, and then placed at 32°C until the MMA cured. Plastic-embedded sections were sectioned to 8 µm on a Leica hard tissue microtome. Staining for the new osteoid formation was done using the traditional Goldner's Masson trichrome stain.

### Statistics

Statistical analysis was performed using the GraphPad Prism 4.0c software. A one-way analysis of variance (ANOVA) with Tukey's multiple comparison post-hoc test was used to verify statistically significant differences among groups. p<0.05 were considered statistically significant. Data is presented as mean±SD.

## Results

### Formation of vascular networks in fibrin hydrogels

We confirmed that capillary networks formed from HUVECs are stable *in vitro* only in the presence of MSCs (**Fig. S1**). Robust networks were observed in confocal microscopy within two weeks of culture in constructs cultured in endothelial growth media (**Fig. S2 – EGM** group) and remained stable throughout the 6 weeks of cultivation. When OM was used at the beginning of the culture period (**Fig. S2 – OM** group), the cells did not form networks. Instead, significant amounts of ‘debris’ from the diI-stained HUVECs were observed by confocal (and used as a pseudo parameter for evaluating HUVEC viability). Cells in the cocktail medium (EGM+OM 1∶1 ratio) formed vascular networks, as evidenced via confocal imaging of the pre-stained HUVECs, however high amount of cell debris was observed (**Fig. S2 – cocktail** group).

Sequential induction was investigated in Group 3 and Group 4 constructs, using EGM first, since HUVECs did not survive well if OM was used first. In both groups, vascular networks that formed within the first two weeks were maintained for the duration of culture. However, supplementation of cocktail medium (EGM+OM 1∶1 ratio) with BMP-2 (10 ng/mL) significantly decreased the amount of cell debris, suggesting that BMP-2 may help maintain the viability of the endothelial cell population (**Fig. S2 – EGM|cocktail+BMP** group).

The amount and pattern of mineral deposition also differed significantly when sequential induction was utilized (Groups 3 and 4) as compared to the osteogenic conditions applied throughout culture (Group 1). Notably, the new mineral deposition was observed mostly in the close spatial proximity to vascular networks, particularly in the **EGM|cocktail+BMP2** group. From these studies, it was determined that sequential induction was needed to facilitate the development of both structures within single constructs for bone tissue engineering applications.

### Formation of bone and capillary networks in scaffolds cultured in vitro

**Figure 2:**
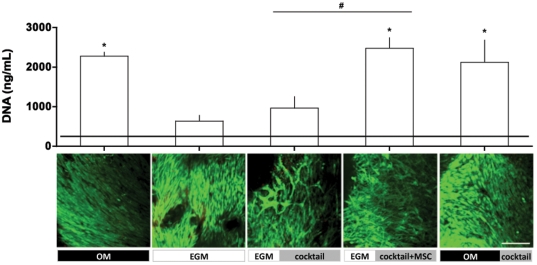
DNA contents of constructs after 6 weeks of *in vitro* culture. **Upper:** Horizontal line indicates day 1 values. n = 3; * indicate p<0.05 in comparison to day 1 values. # indicate p<0.05 among groups. **Lower:** Live/dead imaging of constructs after *in vivo* culture. Scale bar = 200 µm.

Based on the screening studies performed on fibrin gel, we chose to supplement all osteogenic medium with BMP-2 for its role in maintaining the viability of the endothelial population. HUVECs and hMSCs were seeded uniformly throughout the decellularized bone scaffold and located predominantly on the wall surfaces (**Fig. S3**). The DNA content of constructs revealed that **OM** enhanced cell proliferation considerably more than **EGM** (p<0.05). This was clearly a specific cellular response to factors in the **OM** medium since **EGM|cocktail** also had more cells than **EGM** only. Not surprisingly, Group 4 (**EGM|cocktail+MSC**) and Group 5 (**OM|cocktail**) had the most cells (**Fig. 2**), given that more cells were added to these constructs. Confocal microscopy demonstrated cell viability in all groups (Live/Dead assay) and robust capillary networks, in Group 3 and Group 4 (calcein assay) (**Fig. 2**).

**Figure 3:**
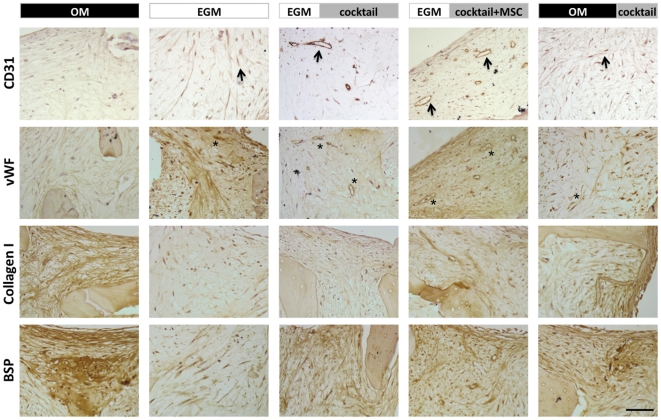
Immunohistological analysis of constructs cultured *in vitro*. Engineered bone grafts were evaluated for the expression of vascular and osteogenic proteins. CD31 (black arrows) and vWF (indicated by *) expression was observed in constructs from all groups. Vascular structures were most developed in **EGM|cocktail** and **EGM|cocktail+MSC** groups. Collagen I and BSP were readily apparent in the **OM** group. Expression was observed in individual cells in EGM group, but not distributed through matrix. Both collagen I and BSP were observed in all other groups. In **EGM|cocktail+MSC** group, BSP appeared in close proximity to the vascular structures. Scale bar = 20 µm.

The two control groups, **OM** (Group 1, osteo-induction) and **EGM** (Group 2, vascular induction) were compared to Groups 3, 4 and 5 where vascular supplements were applied in sequence with osteogenic supplements. Anti-human CD31 and vWF staining demonstrated formation of vascular structures in all groups except for the **OM** (Group 1). Vascular structures were clearly most developed in **EGM|cocktail** (Group 3) and **EGM|cocktail+MSC** (Group 4). While there were CD31 and vWF positive cells in the **OM|cocktail** groups, the vascular networks did not show signs of maturity as evidenced by the sizes of lumens in the structures (**Fig. 3**).

**Figure 4:**
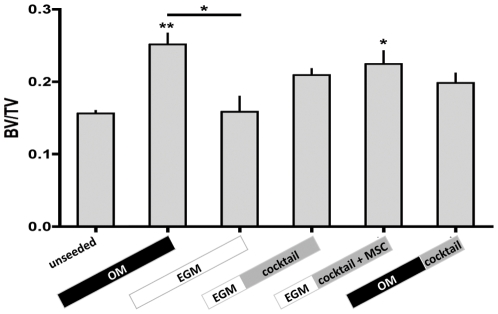
Ratio of bone material volume over tissue volume (BV/TV) of constructs after *in vitro* culture. n = 3; * indicate p<0.05 and ** indicate p<0.001 in comparison to unseeded group. # indicate p<0.05 among both groups.

The **OM** group stained most intensely for collagen I and bone sialoprotein II (BSP), which are indicative of osteogenic differentiation, with incorporation of both collagen and BSP into the new tissue matrix. In contrast, the osteogenic markers in the **EGM** group were confined to individual cells. In the **EGM|cocktail+MSC** group, high expression of BSP was observed in spatial proximity to lumenal structures (**Fig. 3**). BV/TV of tissue constructs, determined by μCT, demonstrated a concordant trend: constructs that were cultured for 6 weeks in osteogenic medium showed the highest BV/TV, while those cultured in endothelial growth medium, show the lowest BV/TV that was close to that observed for unseeded scaffolds. In all other groups, the BV/TV was comparable to that in the **OM** group, being highest for the **EGM|cocktail+MSC** group (**Fig. 4**).

### Properties of capillary networks in vivo

**Figure 5:**
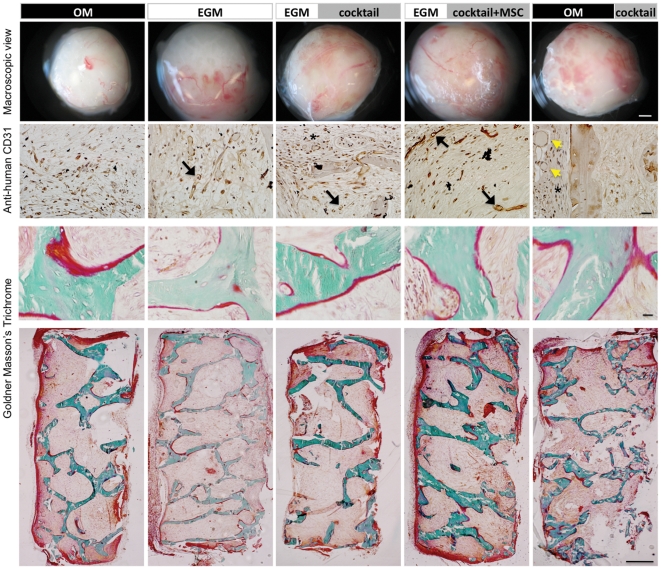
Composition of engineered grafts. **Top row:** Gross images of constructs post-harvest showing the translucent capsule and the in-growth of blood vessels. Constructs are perfused to different extents in different experimental groups. The large blood vessels also seem to anastomose to engineered microvasculature resulting in blood flow through capillary-like networks. This is particularly evident in **EGM|cocktail** and **EGM|cocktail+MSC** groups. Scale bar = 500 µm. **Second row:** Constructs were stained with anti-human CD31. Capsular region filled with mouse cells are shown with asterisks (*). **In EGM|cocktail** and **EGM|cocktail+MSC** groups, lumen (stained with anti-human CD31) are larger and well-developed (black arrows) with red blood cells inside (stained with hematoxylin). In **OM|cocktail** group, there is little evidence of human-derived vascular tissue. Large vessel-like structures in the capsular region are not stained with anti-human CD31 mAb and are probably of mouse origin (yellow arrow heads). Scale bar = 50 µm. **Rows 3 and 4:** *In vivo* bone development. Goldner's Masson trichrome staining of non-decalcified constructs indicate distinct levels of osteoid formation among groups (red stains indicated by black arrow heads). Third row: scale bar = 20 µm, Bottom row: scale bar = 500 µm.

Macroscopic photographs taken with the stereomicroscope indicated in-growth of large, well-developed blood vessels into each of the constructs (**Fig. 5**). In some groups, blood perfusion through microvascular network structures was also observed. Only a few capillaries were seen in the **OM** group, as compared to a higher vascular density in the **EGM** group. Groups 3 and 4, where vasculogenesis was induced prior to osteogenic differentiation, showed the development of the most robust capillary networks. Decalcified constructs were stained with anti-human CD31 to confirm that the vascular networks present prior to implantation remained viable and functional *in vivo*. Positively stained structures were found with cells inside, in all groups where vasculogenesis was induced prior to osteogenesis (Groups 3 and 4) suggesting that the perfused microvasculature might be of human origin (**Fig. 5** and **Fig. S4**). Although there were positively stained cells in the **OM|cocktail** group, these vessels were not well developed and there was no evidence of perfusion. Non-decalcified grafts harvested from *in vivo* studies were stained with Goldner's Masson Trichrome to visualize osteoids, which were observed to a higher extent in the **OM** and **EGM|cocktail+MSC** groups. Clearly, the least osteoid formation was observed in the **EGM** group.

### Proposed model of the development of vascularized bone in vitro

**Figure 6:**
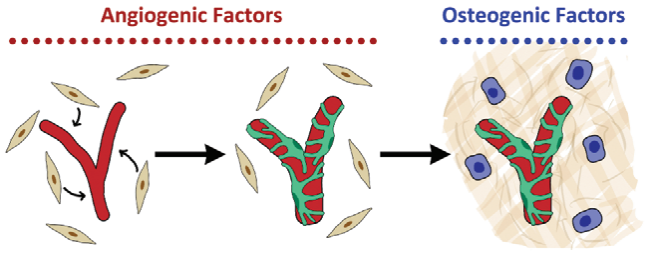
Model of *in vitro* coordination of vascular and bone tissue development. In a co-culture of ECs and hMSCs (beige, spindle-shaped cells), angiogenic supplements provide cues to stimulate the formation of primitive vascular networks (red) by the ECs (dispersed ECs are not shown in the model). These, in turn, recruit hMSCs into a pericyte-like role (green) that enables the vascular network to remain stable when osteogenic cues are provided. These cues induce osteoblast formation (blue cells) and deposition of mineralized matrix (beige).

In initial hydrogel studies, we found that providing osteogenic medium to co-cultures of HUVECs and hMSCs was detrimental to vascular formation. As a result, subsequent studies induced robust vascular development through the application of EGM for two weeks. Early studies demonstrated that the capillary-like structures were formed by HUVECs within a couple days and were only stable in the presence of hMSCs. This suggests that the hMSCs may be playing a pericyte-like role in this system (**Figure 6**). One prevailing view in the literature is that the process of perivascular cell migration and recruitment is mediated by PDGFR signaling [24], [25], [26]. Sequential addition of osteogenic factors to the EGM, and potential BMP expression by EC's [27] facilitated *de novo* bone formation by undifferentiated hMSCs over a period of several weeks. The bone formation was augmented when osteo-induced MSCs were added to the bone scaffolds at the same time as the osteogenic supplements. Moreover, several studies have shown that BMP stimulate VEGF expression in osteoblasts, and VEGF up-regulate BMP-2 mRNA and protein expression in microvascular endothelial cells [27], [28].

## Discussion

The major goal of this study was to test the hypothesis that the sequential application of growth factors, to first induce the formation of stable vasculature and then initiate osteogenic differentiation, could provide a biologically-inspired *in vitro* model of bone vascularization. To this end, we systematically studied cultivation conditions that give rise to both functional vascular structures and robust osteogenesis using HUVEC-MSC co-cultures in conjunction with previously developed tissue engineering and bioreactor methods [19], [20], [29]. We propose that the system used in this study might provide a high-fidelity tissue engineering model of vascularized bone for mechanistic studies of heterotypic cell-cell interactions between endothelial cells and osteo-progenitors. In fact, a recent study showing that vascular endothelial cells may possess an intrinsic capability of transitioning to mesenchymal progenitors [30] opens tremendous possibilities for the role of endothelial cells in this model.

In our model, the MSCs have two major roles: as pericytes stabilizing vascular networks and as osteogenic progenitors forming mineralized bone matrix. Several published studies show that bone marrow-derived MSCs adopt pericyte-like locations during *in vitro* co-culture with mature endothelial cells [15], [31], [32] or when co-delivered *in vivo* with mature endothelial cells [24], [33], [34]. Our study supports these previous observations as the capillary-like structures formed by HUVECs were only stable in the presence of hMSCs (**Fig. S1**).

The ability of MSCs to respond with spatial and temporal specificity to differentiation cues was key in gel studies where all cells were seeded at the beginning of culture and no cells could be introduced at later stages of culture (supplemental data). From these preliminary studies, several key outcomes were identified that determined the selection of experimental groups for the subsequent scaffold study: (i) a sequential-induction approach enabled vascular-osteogenic outcomes within the same culture space, with the need for endothelial growth factors to be provided prior to osteogenic induction, and (ii) the role of BMPs as a vascular promoter was confirmed [35] even during *in vitro* culture. Mineralization of the tissue matrix was evident in the groups containing vascular networks, although to limited extent as cells were exposed to osteo-inductive medium for only two weeks. Thus, a longer osteo-induction period was adopted during scaffold studies.

Trabecular bone scaffolds also provided great versatility of the *in vitro* co-culture system and enabled the evaluation of concurrent supplementation of growth factors and fresh MSCs responding to these growth factors. We also investigated the hypothesis of MSCs losing their ability to respond to osteogenic factors during the two-week exposure to vascular supplements, by comparing Group 4 (**EGM|cocktail+MSC**, in which osteo-induced MSCs were added along with cocktail medium) with the Group 3 (**EGM|cocktail**, in which the same MSCs present at the beginning of culture were exposed to cocktail medium). Data suggests that fresh MSCs enhance bone formation, as verified by increased bone volume fraction (Fig. 4), osteoid deposition (Fig. 5), and spatial proximity of bone proteins to vascular networks (Fig. 3). However the addition of additional MSCs is not critical, as bone formation also occurred in Group 3 (**EGM|cocktail**). This suggests that MSCs may act initially as pericytes [15], [31], [32], but maintain the ability to undergo osteogenesis, which is arguably consistent with the prior evidence that osteoblasts take up pericytic locations *in vivo* [3].

By adding HUVECs at the latter stages of culture, it was possible to evaluate osteogenesis that has been induced in the scaffolds prior to providing vascular cells and growth factors, an approach similar to that taken by Tsigkou et al [15]. This approach to improve vascularized bone tissue development (**OM|cocktail** group) was moderately successful: we observed reasonable osteogenic development as evidenced by deposition of bone protein, osteoid formation and the BV/TV ratio. However, two-week exposure to vascular growth factors was not sufficient to develop robust vascular structures, as evidenced by the sizes of vessel lumens that were smaller than those observed in Groups 3 and 4. In these two groups, not only lumen were evident after *in vitro* cultivation, they also anastomosed well during *in vivo* implantation, which is consistent with the earlier reports that well-developed vascular networks are necessary for functional anastomosis [36], [37], [38], [39], although recent studies suggest that is also possible to obtain functional network formation without extensive *in vitro* cultivation [8], [15]. These studies open several under-explored areas of investigation. For example, the DNA content was considerably higher in the OM group relative to the EGM group, suggesting that HUVECs may have survived better as a result of BMP2 supplementation in osteogenic media (**Fig. 4**).

In future studies, it might be relevant to distinguish whether HUVECs and MSCs are equally stimulated in this group and to decipher the role of cell proliferation in tissue development. Also, despite promising results, our studies suggest that alternative cocktails (e.g. EGM|OM or increased vascular growth factor concentration in OM|cocktail group) could be tested to synergistically improve mineralization with vascular network formation. Likewise, further studies are needed to optimize the temporal stages of culture, as the two-week period of vascular induction was selected based on the robust network structures observed for fibrin gel studies.

Overall, by providing temporal variation of the culture environment, we were able to demonstrate the ability to derive vascular networks and induce *bona fide* osteogenic differentiation within single tissue grafts during *in vitro* cultivation. Notably, functional vascular networks and viable osteoid formation were detected post-implantation. The most successful approach involved induction of vasculogenesis prior to osteogenesis, with the addition of osteo-induced MSC further improving tissue outcomes. Finally, while the focus of our study was to evaluate ‘functional’ outcomes such as vessel formation and matrix mineralization, significantly more work is needed to elucidate the underlying mechanisms that regulate heterotypic interactions and understand the interactive roles of multiple growth factors.

In summary, it has long been accepted that vascularization is essential for the advancement of tissue-engineered bone grafts to clinical application [40]. Due to the close interaction between MSCs and HUVECs in co-culture systems, it may be possible to utilize the proposed tissue engineering model as a controllable experimental tool to elucidate other molecular mechanisms regulating the heterotypic cell-cell interactions. Such studies could have relevance for gaining further insights into developmental processes [41] and cellular interactions under conditions of disease.

## Supporting Information

Figure S1 **Gel cultivation.** **A:** HUVECs only at 3 weeks in EGM-2. Co-culture of HUVECs with MSCs (1∶1) enables formation of stable micro-vasculature networks at 3 weeks in EGM-2 (**B**) that last up to 12 weeks (**C**) during *in vitro* culture. **D:** von Willebrand Factor (vWF) staining of lumen.
(JPG)

Figure S2 **Gel screening studies.** HUVECs (pre-stained with Di-I) and MSCs were encapsulated in fibrin hydrogels (1∶1 ratio) and cultured in small wells for 4 weeks to determine cellular responses to different medium conditions. Confocal images were used to evaluate vascular network formation and provide a read-out on HUVEC viability (cellular debris). Scale bar = 50 µm. **Bottom row:** von Kossa staining of mineral deposition within the gel regions. Mineral is shown as black/dark brown stains within the gels.
(JPG)

Figure S3 **H&E staining of constructs at day 1.** Cells are uniformly distributed throughout the scaffold upon seeding. Cells are located predominantly on the wall surfaces of scaffolds but grow into pore spaces subsequently.
(JPG)

Figure S4 **EGM|cocktail+MSC group stained with anti-human CD31 mAb.** Human origin lumen, with red blood cells inside (stained with hematoxylin) are pointed with yellow arrows.
(JPG)
