# Supplementary figures and images for: In Vitro Model of Vascularized Bone: Synergizing Vascular Development and Osteogenesis
Source: PLoS One. 2011 Dec 2;6(12):e28352. doi: 10.1371/journal.pone.0028352 (PMC3229596; doi:10.1371/journal.pone.0028352)

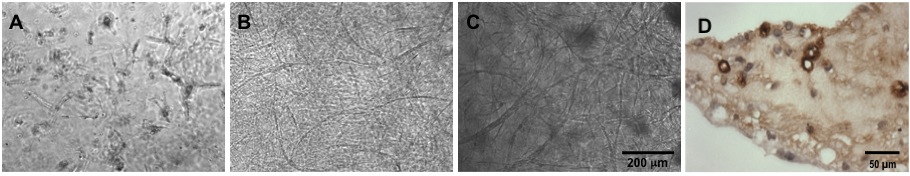

Supplement: Figure S1 — Gel cultivation. A: HUVECs only at 3 weeks in EGM-2. Co-culture of HUVECs with MSCs (1∶1) enables formation of stable micro-vasculature networks at 3 weeks in EGM-2 (B) that last up to 12 weeks (C) during in vitro culture. D: von Willebrand Factor (vWF) staining of lumen. (JPG) [file pone.0028352.s001.jpg]

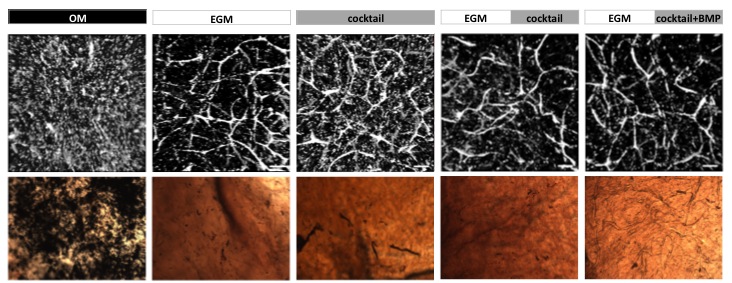

Supplement: Figure S2 — Gel screening studies. HUVECs (pre-stained with Di-I) and MSCs were encapsulated in fibrin hydrogels (1∶1 ratio) and cultured in small wells for 4 weeks to determine cellular responses to different medium conditions. Confocal images were used to evaluate vascular network formation and provide a read-out on HUVEC viability (cellular debris). Scale bar = 50 µm. Bottom row: von Kossa staining of mineral deposition within the gel regions. Mineral is shown as black/dark brown stains within the gels. (JPG) [file pone.0028352.s002.jpg]

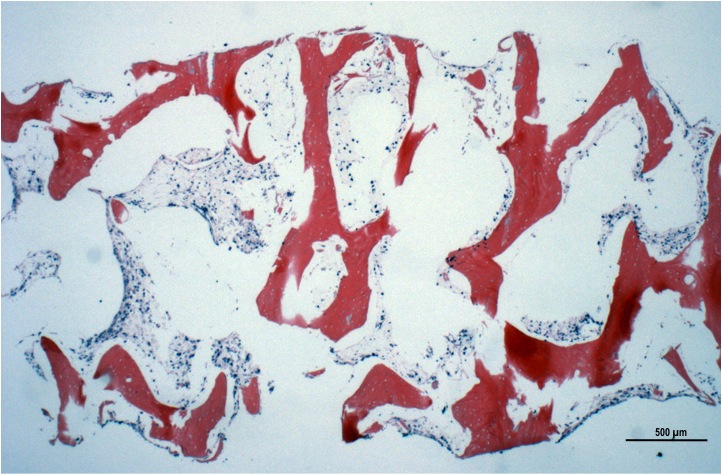

Supplement: Figure S3 — H&E staining of constructs at day 1. Cells are uniformly distributed throughout the scaffold upon seeding. Cells are located predominantly on the wall surfaces of scaffolds but grow into pore spaces subsequently. (JPG) [file pone.0028352.s003.jpg]

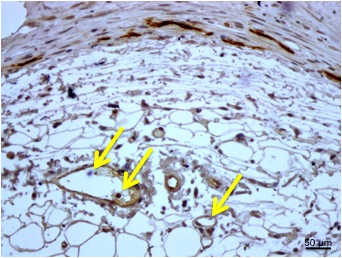

Supplement: Figure S4 — EGM|cocktail+MSC group stained with anti-human CD31 mAb. Human origin lumen, with red blood cells inside (stained with hematoxylin) are pointed with yellow arrows. (JPG) [file pone.0028352.s004.jpg]
